# *In silico* design and expression of a novel fusion protein of HBHA and high antigenic region of FAP-P of *Mycobacterium avium* subsp. *paratuberculosis* in *Pichia pastoris*

**DOI:** 10.22099/mbrc.2017.26522.1286

**Published:** 2017-12

**Authors:** Vida Eraghi, Abdollah Derakhshandeh, Arsalan Hosseini, Azar Motamedi-Boroojeni

**Affiliations:** Department of Pathobiology, School of Veterinary Medicine, Shiraz University, Shiraz, Iran

**Keywords:** *FAP-P*, fusion protein, HBHA, *in silico*, *Mycobacterium avium* subsp

## Abstract

*Mycobacterium avium *subsp. *paratuberculosis* (MAP) is the etiologic agent of Johne's disease in ruminants and there has been a shift in the public health approach to MAP and human diseases like Crohn's disease. The prevention of infection by MAP in ruminants is thought to deter the high impact of economic losses in the level of dairy industry and possible spreading of this pathogen in dairy products. The present study was done to investigate the construction and expression of the soluble form of a novel fusion protein, consisting of Heparin-binding hemagglutinin (HBHA) and high antigenic region of Fibronectin Attachment Protein-P (FAP-P), in order to introduce as a Th1 inducer subunit vaccine against MAP. HBHA is a mycobacterial adhesin and it has been demonstrated that a HBHA-specific IFN-γ response, in latent *M. tuberculosis* infection, depends on the methylation of the antigen. Further, *FAP-P* induces Th1 polarization. Because methylation of HBHA was not performed in *E. coli*, *Pichia pastoris* was chosen as the host. The desired fusion protein had a similar 3D structure to that of HBHA with its native form and post-translational methylation in C-terminal. Hence, the uptake of the purified fusion protein will be done by M cells because of HBHA, and cell-mediated immunity will be induced because of both antigens. Eventually, successful construction and expression of the newly-designed chimeric protein under the mentioned conditions is reported in this article.

## Introduction

*Mycobacterium avium *subsp. *paratuberculosis* (MAP) is the etiologic agent of Johne's disease that infects both domestic and wild ruminant animals. The important effect of this disease is its economic significance in the level of dairy industry [1, 2]. There is some similarity between Crohn’s disease in human and its reported association with MAP [3]. In addition to Crohn's, the association of MAP with sarcoidosis and Blau syndrome [4], type 1 diabetes [5], Hashimoto’s thyroiditis [6], and multiple sclerosis (MS) [7] were studied. Thus, MAP can be introduced as a public health concern. Vaccination can be the best control strategy and more cost-effective than testing and culling [8]. Until now, a number of whole-cell based vaccines, live attenuated vaccines, and inactivated vaccines were developed to prevent bovine and ovine Johne’s disease [9]. Subunit vaccines consist of proteins or DNA encoding immunogenic antigens, and many genomic or proteomic analyses have been carried out to identify MAP antigen. IFN-γ induced by Th_1_-mediated immune responses is crucial to reducing the number of bacteria in the early stages of Johne's disease. Next, it is the induced strong Th1 responses which are essential to the development of subunit vaccines [10]. MAP exploited M cell function to cross the intestinal barrier and enter into the supepithelium [11]. Heparin-binding hemagglutinin (HBHA) is an important mycobacterial adhesin on the surface of bacteria and it has interaction with sulfated glycoconjugates present on the surface of the host cells [1, 11]. This antigen induces humoral immune responses from the host during bovine tuberculosis and Johne's disease. Native HBHA from *M. tuberculosis* has post-translational modification consisting of methylation of lysines in the C-terminal domain [12] and the methylation of HBHA which is responsible for specific IFN-γ response in latent *M. tuberculosis* infection [13, 14]. Fibronectin attachment proteins (FAPs) are important for attachment and internalization of MAP by epithelial cells in vitro [15]. FAP induces dendritic cells to increase the production of IL-12 and subsequently Th_1_ polarization and IFN-γ production by T cells in vitro [16]. Despite the obvious advantages of bacterial expression systems such as *Escherichia coli* and *Pichia pastoris*, methylotrophic yeast with high level of production of recombinant proteins is an important diverse post-translational modification for HBHA, including polypeptide folding and methylation. In addition, growing to high cell densities has become an interesting and important alternative to bacterial expression systems [17]. *P. pastoris* (strain GS115) expression system is employed in the expression of authentic heterologous proteins of high quality and high quantity to be secreted in a soluble form [18, 19]. Strain GS115 is defective in the histidine dehydrogenase gene (his4), and pPIC9K contains histidine dehydrogenase gene (HIS4). Thus, growing on a non-histidine containing media can be used as a selectable marker for transformants [18]. Based on these data, we chose HBHA and high antigenic region of FAP-P to have adhesion and activation of cell-mediated immunity. We constructed and expressed a newly designed fusion protein in* Pichia pastoris* as a host because methylation of HBHA was not performed in *E. coli*, and we were able to confirm secretory expression of a soluble form of chimeric protein with an expected size of 32.9 kDa in culture media using western blot.

## MATERIALS AND METHODS

**pPICK9KHBHA-FAP construction and cloning procedure: **Sequences registered at NCBI with accession numbers of KF021287 for *FAP-P* gene (990 bp) and KC920678 for *HBHA* gene (618 bp) were analyzed. To design fusion gene, an encoded protein of FAP-P was assessed by CLC software (main workbench 5) as to determine high antigenic part among the full-length sequence of the gene. For better folding of *HBHA*, rigid linkers group was chosen to be introduced between two genes [20, 21]. In order to evaluate the secondary structure and determine the best number and composition of linker amino acids, each protein was assessed by CLC software before and after the fusion with a linker. Protein Homology/analogY Recognition Engine (Phyre2) (http://www.sbg.bio.ic.ac.uk/~phyre2/html/ page.cgi?id=index) was used for obtaining PDB (protein data bank) files of three-dimensional structure of HBHA and chimeric protein. Afterward, TM-align was used for analyzing the alignment of three-dimensional structure of the chimeric protein with native HBHA based on achieved TM-score (http://zhanglab.ccmb.med.umich.edu/TM-align) [22].

Codon optimization of nucleotide sequence of the fusion gene was done for increasing the expression level of the desired protein in *Pichia pastoris*, using codon optimization tool (https://eu.idtdna.com/CodonOpt). The *Pichia pastoris* codon usage table was adapted from a codon usage database (http://www.kazusa.or.jp/codon/cgi-bin/showcodon.cgi?species=4922). Full-length codon optimized HBHA-FAP gene in pPICK9K yeast transfer vector was synthesized by Biomatik company (Canada), so the gene fragment was located between *Eco*RI and *Not*I cut sites under AOX1 promoter in fusion with *S. cervecea* alpha secretory signal at N-terminus. *Pichia pastoris* strain GS115 (obtained from Pasteur Institute, Iran) was selected as the expression host and was grown at 28°C in YPD agar medium (including Yeast Extract, Peptone, Dextrose, 2% Agar). 

For cloning procedures, *E. coli* DH5α was grown at 37°C in Luria-Bertani medium (Himedia, India). The synthetic pPICK9KHBHA-FAP was transformed into *E.coli* DH5α strain via chemical transformation method. Recombinant transformants were selected by culturing in LB medium containing 50μg/ml ampicillin. Digestion of plasmid DNA using *Eco*RI and *Not*I enzymes (Roche, Germany) was done for confirmation of positive bacterial transformants.

**Expression analysis and optimization of HBHA-FAP expression: **The recombinant plasmid pPICK9KHBHA-FAP was linearized by digesting it with *Sac*I enzyme (Roche, Germany) in order to integrate the transgene at His4 locus in the *P. pastoris* genome and generating HIS^+^, Mut^+^ transformants. Ten micrograms of the linear DNA were used to be transformed into fresh electrocompetent *P. pastoris* cells. Electroporation was performed using Bio-Rad Gene Pulsar Xcell™ electroporation system (Bio-Rad Laboratories, Inc USA) with a voltage of 2000V, 25μF capacitance and 200Ω resistance. The electroporated cells were immediately diluted in 1 mL of ice-cold 1 M sorbitol. 200-µL aliquots were spread on MD plates (including Yeast Nitrogen Base medium wo/AA, Dextrose, Sorbitol, Biotin, Agar) and incubated at 30°C for 3 days. For negative control, pPIC9K without insert, linearized with *Sac*I, was also transformed similarly. The colonies obtained were streaked on fresh MD plates.

The glycerol stocks of the positive transformants of *P. pastoris*, along with the negative control (*P. pastoris* transformed with pPICK9K without insert), was inoculated separately into 25 ml of Buffered Minimal Glycerol medium-BMGY (1% yeast extract, 2% peptone, 100 mM potassium phosphate buffer pH 6.0, 1.34% yeast nitrogen base without amino acids (YNB), 4×10^-5^% biotin, 1% glycerol) taken in 250 ml conical flask, and was incubated at 30°C in a shaker incubator at 250 rpm until the A600nm of the culture reached 2-6. The cells were harvested by centrifugation at 3,000 × g for 15 min at room temperature and the cell pellets were resuspended in the required volume of Buffered Minimal Methanol medium-BMMY (BMGY that contains 1% methanol instead of glycerol) to get an A600nm of 1 in 1000 ml conical flasks. Three methanol concentrations (0.5, 1.0, and 1.5%) for induction of expression were tried in both media in order to determine the optimum concentration of methanol. Incubation was continued in the orbital shaker incubator (30°C, 250 rpm) for five days. To maintain the induction, methanol was added to culture the media (0.5% v/v) once in every 24 hours after collecting culture supernatants.

The culture supernatants under optimal expression conditions were collected. The secreted protein was removed by pelleting the cells, and an equal volume of 20% trichloroacetic acid was added to the supernatant. The mixture was incubated overnight at -20°C. For harvesting the protein precipitate, the mixture was centrifuged at 12,000g for 30 min, and washing of the pellet was done with acetone. Eventually, the resulting protein was resuspended in phosphate-buffered saline (PBS) for next steps.

**SDS-PAGE and** W**estern blot analysis of fusion protein: **The concentrated proteins were analyzed by running them on 12% polyacrylamide gel electrophoresis under denaturing condition. Then, the gels were stained with Coomassie Brilliant Blue R-250 (Merck, Germany). Western blotting was performed for identification of the histidine tag expressed of the recombinant fusion protein. Hence, the separated proteins by SDS-PAGE were electrotransferred onto a nitrocellulose membrane and incubated with a 1:1000 dilution of monoclonal anti-polyhistidine-peroxidase (Sigma, USA). The color developing was done using H_2_O_2_/DAB substrate/ chromogen (Sigma, USA).

## RESULTS

**Figure 1:**
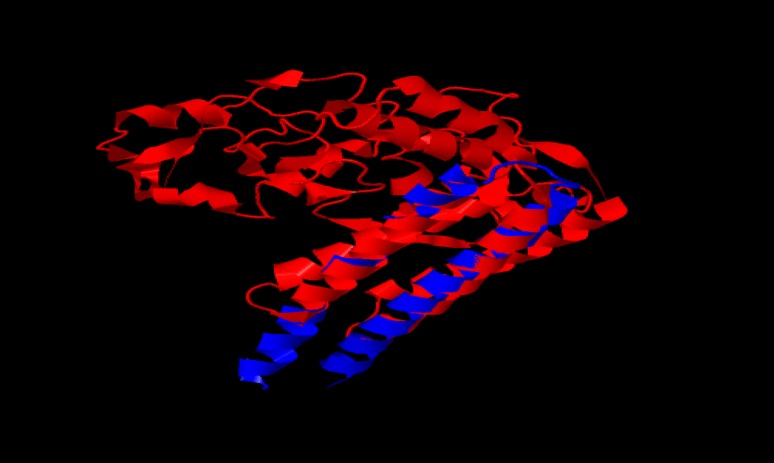
3D structure alignment of chimeric protein with native form of HBHA. Alignment of HBHA-FAP (in red) with native HBHA (in blue).

The most antigenic region of *FAP-P* from amino acid 125 to 205 (240 bp of the gene) and the entire gene of *HBHA* were selected. For the evaluation of the secondary structure and selection of appropriate linker, amino acid sequences of HBHA and fusion protein were assessed before and after the fusion with a linker. At least (Pro Glu)_7_ were used as linker. Three-dimensional structure of HBHA and the chimeric protein of fusion gene were obtained as PDB and the similarity index between HBHA domain of chimeric protein and its native structure was obtained by TM-score measurement (Fig. 1). Eventually, codon-optimized fusion gene as an insert in pPIC9K between restriction sites of *Eco*RI and *Not*I was synthesized.

The electroporated resultant pPICK9KHBHA-FAP, linearized with *Sac*I restriction enzyme into *P. pastoris*, yielded His^+^ Mut^+^ transformants per 10μg of DNA used and they could grow on histidine-deficient medium. The absence of a yeast origin of replication in the plasmid assured that transformed yeast carried the plasmid integrated into the yeast DNA. Transformed yeast colonies appeared in 3 days at 30°C. 

**Figure 2:**
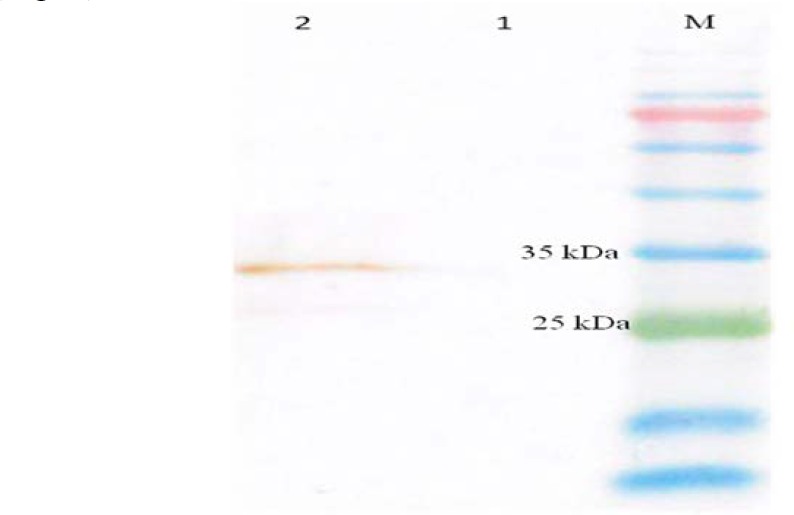
**Western blot analysis of recombinant protein** **.** Lane M: protein ladder (Cinnagen PR911654 [SL7012 ]), Lane 1: negative control, Lane 2: recombinant HBHA-FAP protein 120 hr of post-induction with 0.5% methanol concentration with the same expected size.

*S. cerevisiae* α-mating factor pre-pro leader sequence secretory signal (SS) in the pPICK9KHBHA-FAP construct was employed, resulting in the release of the matured, fully processed HBHA-FAP protein. Some of the His^+^ Mut^+^ colonies that were found positive through selecting media were subcultured and definitely positive samples were stored in -80°C with glycerol. Three colonies were selected for inducing the expression of target gene. Expression of HBHA-FAP recombinant protein, with a size of ~35 KDa, was detected in two clones after 72 hr of induction. The best expression level was obtained 120 hr post-induction with 0.5% methanol concentration (Data not shown). With the above optimized expression conditions, properly folded -FAP was secreted into the medium as a soluble protein. Western blotting using HRP-anti-polyhistidine (Sigma, USA) confirmed the protein expression. Proper positive signals were obtained only in culture supernatants of positive yeast transformants, not with pPICK9K negative control (Fig. 2).

## DISCUSSION

To control paratuberclosis, developing new vaccines that confer effective protection against MAP is useful. Many genomic or proteomic analyses have been done to identify MAP antigens for developing subunit vaccines. Until now, several antigens of MAP have been introduced as a vaccine candidate. Some of them were tested for their potential to be used as a vaccine candidate including heat shock protein 70 (Hsp70) [23], antigen 85 complex proteins (Ag85A, Ag85B, and Ag85C) [24], lipoproteins (LprG and MAP0261c) [25, 26], PPE family proteins (MAP1518 and MAP3184) [27], superoxide dismutase [24], and alkyl hydroperoxide reductases (AhpC, AhpD) [28]. In this investigation, two antigens based on their ability in adhesion and activation of cell-mediated immunity were selected. HBHA is a major adhesin of MAP that can interact with receptors on the surface of eukaryotic target cells [29]. Native HBHA from *M. tuberculosis* has post-translational modification consisting of mono- and dimethylation of lysine in the C-terminal, which is important for its antigenicity [12]. It has been demonstrated in several studies that methylated HBHA produces specific IFN-γ response in latent *M. tuberculosis* infection [13, 14]. This post-translational modifica-tion not performed using *E. coli* as a host [30].

FAP-P interacts with FN for attachment and internalization of MAP by epithelial cells, and induces Th1 polarization and IFN-γ production by T cells in vitro [16]. FAP has an immunostimulatory ability and it can be utilized for increasing immunogenicity [31]. At the first time, FAP-P (ModD) homologue was evaluated in *M. tuberculosis* and *M. bovis* and reported to be a dominant secreted antigen [32, 33]. Also, it has been identified as a major secreted protein within mycobacteria-containing phagosomes [34]. Previous studies have shown that the infected macrophages by *M. avium* that presented FAP peptide to T cells contribute to the development of protective immunity to this antigen [16, 35]. In the early stages of Johne's disease, IFN-γ induced by Th1-mediated immune responses is crucial to reducing the number of bacteria. Also, inducing strong Th1 responses is essential to the development of subunit vaccines [10]. Using western blot analysis, HBHA-FAP recombinant protein had a size close to 35 KDa marker band. Expected size (32.9 KD) was determined based on our *in silico* analysis and the reports on the molecular size of *HBHA* in previous studies [11, 29].

To date, researchers have focused on cell-mediated immune responses to identify candidate vaccines and in this investigation, a new fusion protein consisting of HBHA and high antigenic region of FAP-P was introduced. Based on *in silico* analysis and choosing *P. pastoris* as a host, the desired fusion protein had a similar 3D structure to that of HBHA with its native form and post-translational methylation in C-terminal. Hence, the uptake of the purified fusion protein is done by M cells because of HBHA being an adhesion, and cell-mediated immunity is induced against both antigens. Checking the potential of the designed fusion protein in activation of cell-mediated immunity in animal models is being done in our laboratory.
